# Mechanisms of antidepressant resistance

**DOI:** 10.3389/fphar.2013.00146

**Published:** 2013-11-22

**Authors:** Wissam El-Hage, Samuel Leman, Vincent Camus, Catherine Belzung

**Affiliations:** ^1^INSERM 930, Faculté de Sciences et Techniques, Université François RabelaisTours, France; ^2^Centre Hospitalier Régional Universitaire de Tours, Centre Expert Dépression Résistante, Fondation FondaMentalTours, France

**Keywords:** major depression, resistance, antidepressants, treatment-resistant depression, monoamine

## Abstract

Depression is one of the most frequent and severe mental disorder. Since the discovery of antidepressant (AD) properties of the imipramine and then after of other tricyclic compounds, several classes of psychotropic drugs have shown be effective in treating major depressive disorder (MDD). However, there is a wide range of variability in response to ADs that might lead to non response or partial response or in increased rate of relapse or recurrence. The mechanisms of response to AD therapy are poorly understood, and few biomarkers are available than can predict response to pharmacotherapy. Here, we will first review markers that can be used to predict response to pharmacotherapy, such as markers of drug metabolism or blood-brain barrier (BBB) function, the activity of specific brain areas or neurotransmitter systems, hormonal dysregulations or plasticity, and related molecular targets. We will describe both clinical and preclinical studies and describe factors that might affect the expression of these markers, including environmental or genetic factors and comorbidities. This information will permit us to suggest practical recommendations and innovative treatment strategies to improve therapeutic outcomes.

## Introduction

Major depressive disorder (MDD) is among the most frequent mental disorders, with an estimated lifetime prevalence of 1–16%, depending on the country (Andrade et al., 2003; Kessler and Ustun, 2004; Kessler et al., 2010). Recovering from MDD is a major challenge because the disease dramatically increases the risk of suicide (Cheng et al., 2000) and non-suicide mortality (Schulz et al., 2002). Practical guidelines recommend treating MDD with antidepressant (AD) therapy (NICE, 2004; Bauer et al., 2007; Ramasubbu et al., 2012). Since the initial serendipitous discovery of the AD effect of monoamine oxidase inhibitors (MAOIs) and tricyclics (TCAs), most ADs have been pharmacological agents that act on monoamine function, including serotonin (selective serotonin reuptake inhibitors, SSRIs), noradrenaline (noradrenaline reuptake inhibitors, NRIs), dopamine (such as bupropion), and melatonin (agomelatine). Some drugs act on several of these targets (serotonin and noradrenaline reuptake inhibitors, SNRIs) (Krishnan and Nestler, 2008). The main goals of treating MDD are to achieve remission and to maintain these therapeutic effects over time. In the absence of any reliable biomarker of MDD, the response to treatment is still based on clinical assessment as evidenced by changes on scores on standardized rating instruments, such as the Hamilton Depression Rating Scale (HDRS) (Hamilton, 1960) or the Montgomery Asberg Depression Rating Scale (Montgomery and Asberg, 1979). The response to ADs is typically characterized as “non-response” when only minimum improvement is achieved, “partial response” when the score on the standardized instrument decreases by 25–50%, “response” when a decrease of at least 50% is obtained, and “remission” when only residual clinical symptoms are reported, with a level of psychopathology under the typical threshold score currently correlated to MDD diagnosis (Nierenberg and DeCecco, 2001). Using these criteria, several studies, including the naturalistic STAR^*^D study, have shown that only one third of MDD patients receiving ADs achieve complete remission after a single AD trial (Trivedi et al., 2006). The remission rate reaches up to 60% after four trials, but the probability of remission drops significantly after the failure of two consecutive AD trials (Rush et al., 2006). Moreover, early improvement predicts sustained response and remission (Lam, 2012). Consequently, the concept of treatment-resistant depression (TRD) was proposed to describe depressive conditions that did not reach sufficient remission after treatment (Lehmann, 1974; Sartorius, 1974). Even several criteria have been proposed to define TRD-including non-response to one AD for at least 4 weeks or failure to respond to multiple trials of different classes of ADs-there is now an emerging consensus to consider any MDD patient that did not respond to two or more adequate (in terms of duration and dosage) AD trials of different classes as TRD (Berlim and Turecki, 2007). The characterization of TRD has been improved by considering the level of resistance (severity and duration) through staging classifications, such as the Antidepressant Treatment History Form (ATHF) (Sackeim et al., 1990), the Thase and Rush Model (Thase and Rush, 1997), the European Staging Model (Souery et al., 1999), the Massachusetts General Hospital Staging Model (MGH-s) (Fava, 2003), and the Maudsley Staging Model (MSM) (Fekadu et al., 2009) (for a review, see Ruhé et al., 2012).

Translational research enables to study the mechanisms of non response to ADs in animal models. Indeed, this involves invasive protocols, as particular proteins, brain areas or process have to be suppressed to elucidate their causal involvement in response to AD. Therefore, bioassays (forced swim test, tail suspension tests…) and more generally animal models have been designed that enable to induce a behavioral deficit after experimental manipulations (stressors during the developmental period, social defeat or unpredictable chronic stress during adulthood, chronic corticosterone). The induced modifications are then assessed via behavioral testing such as scoring of sucrose preference, of coat state, of grooming behavior, of anxiety-related behavior. It is usually observed that chronic ADs reverse the behavioral alterations that have been induced by the experimental manipulations and this enables to assess AD response (depending on the magnitude of the reversal that has been observed, and on the number of behavioral dimensions that have been counteracted). It is pivotal here to assess several different behaviors, as it can succeed that only a specific phenotype is reversed, and not the others as seen for example in David et al. (2009).

The main aim of this paper is to review the different potential predictors of response/non response to AD and discuss their clinical and practical implications. We will successively discuss the potential mechanisms and correlates of response to ADs with regard to some general clinical and pharmacological considerations and at the different levels of neurobiological understanding of AD mechanisms of action (Kupfer et al., 2012), with a particular consideration of the neuroanatomical, neurotransmission, molecular, and genetic levels, as well as to potential hormonal and neuroplasticity aspects (Table 1).

**Table 1:** **Mechanisms predicting response to antidepressants**.

**Main predictors of poor response to antidepressant treatment**	
**CLINICAL CORRELATES**	
	Bipolar depression Older age in relation to age or somatic comorbidities (cardiac, cerebrovascular, neurodegenerative disorders) Poor compliance to antidepressants in relation to low income, health insurance status, race/ethnicity
**PHARMACOLOGICAL CORRELATES**	
Drug metabolism	Younger age, sex, smoking status, pregnancy, drug dose, diet, grapefruit, genetics, enzyme induction/inhibition Ultra-rapid metabolizers in relation to hepatic metabolism: genetic differences in drug-metabolizing enzymes (cytochromes P450; e.g. CYP2D62, CYP2C19) Alteration of hepatic, renal or cardiovascular functions Polypharmacy enhances drug interactions, particularly fluvoxamine, fluoxetine, paroxetine, nefazodone
Blood-brain barrier	Polymorphisms in genes coding for ABC transporter proteins, particularly the P-glycoprotein (P-gp) Drugs that are substrates of P-gp have decreased penetration into the brain
**NEUROBIOLOGICAL CORRELATES**	
Brain structures	EEG (alpha and theta activities) Lower alpha rhythmic activity in posterior regions (among amitriptyline non-responders) and in left hemisphere (SSRIs) Higher theta rhythmic activity among imipramine non-responders Decreased pre-treatment theta activity in the ACC
	Neuroimaging (fMRI, PET) Lower baseline rostral ACC activity Low ACC activity during functional tasks Reduction in frontolimbic gray matter volumes (medial and orbital PFC) Smaller baseline hippocampal volume Abnormalities in corticolimbic connectivity Higher right- over left hemisphere processing Higher baseline metabolism in the amygdala and thalamus, and lower pretreatment metabolism in the medial PFC Insula hypometabolism
Neurotransmission	Serotoninergic system Alteration of the 5-HT_1A_ pre- and postsynaptic receptors dynamic Polymorphism of the 5-HT transporter gene (short allele carriers) SNPs of tryptophan hydroxylase genes (TPH1 and TPH2) SNPs of the 5-HT1A receptor gene (1019C/G; 102T/C; 1438A/G) Interaction between stressful life events and polymorphisms in 5-HT related genes
	Noradrenergic system Alteration of the dopamine beta-hydroxylase enzyme/gene Deficiency in organic cation transporter 2 Polymorphisms of the noradrenaline transporter gene (-182T/C; 1287G/A) Polymorphism of the catechol-O-methyltransferase gene (Val homozygous) Early life stress events (via gene methylation or acetylation)
	Other systems Decreased substance P in the cerebrospinal fluid SNPs of the dystrobrevin-binding protein 1 gene (glutamatergic neurotransmission) SNPs of the glutamate receptor ionotropic kainite 4 gene (rs1954787; rs12800734) Deletion of the gene encoding the GABA transporter subtype 1 Genetic variability in endocannabinoid receptors (CNR1; G allele of rs1049353 in females) Deficit in the leptin system (decreased leptin serum levels, reduced leptin mRNA expression)
Neural plasticity	Molecular aspects Polymorphism in the BDNF gene (Val allele carriers) Alteration of BDNF in the dentate gyrus (hippocampus) Alteration of protein p11, mediating the antidepressant activity of BDNF Interaction between ongoing stress and the levels of BDNF Zinc deficiency Macrophage migration inhibitory factor deficiency
	Cellular targets Alteration in adult hippocampal neurogenesis Alteration of the generation of new functional neurons
Hormonal targets	HPA axis Defect in the HPA axis regulation (defect in normalization of its overactivity) No reduction of the cortisol response to a dexamethasone/CRH test after 2–3 weeks of treatment Polymorphisms of genes coding for FKBP5, BclI, ER22/23EK, CRHR1 (rs242941), CRHR2 (rs2270007), CRH-BP, and hsp70 protein Somatic condition: Cushing's disease Interaction between stressors and genes (SERT, FKBP5, CRHR1) to predict response to treatment
	Thyrotropin releasing hormone Hypothyroidism; Polymorphism of the deiodinase type 1 gene

*ACC, Anterior cingulate cortex; BclI, ER22/23EK, Polymorphisms of glucocorticoid receptor gene; BDNF, Brain-derived neurotrophic factor; CRH, Corticotropin-releasing hormone; CRHR-BP, CRH binding protein; CRHR1, CRH receptor 1; CRHR2, CRH receptor 2; EEG, Electroencephalography; FKBP5, FK506 binding protein 5; fMRI, Functional magnetic resonance imaging; HPA, Hypothalamic-pituitary-adrenal; PET, Positron emission tomography; PFC, Prefrontal cortex; SERT, serotonin transporter; SNPs, Single-nucleotide polymorphisms; SSRIs, Selective serotonin reuptake inhibitors; 5-HT, Serotonin*.

## Poor response to antidepressant therapy: clinical correlates

The efficacy of ADs has been strongly debated recently because of negative randomized controlled studies in which the response rates in the placebo control group were as high as 30–40% (Iovieno and Papakostas, 2012). Rather than arguing against the efficacy of ADs in MDD, these results indicated some methodological limitations of studies recently submitted to the European or US authorities (Khin et al., 2011). In particular, an inappropriately low baseline disease severity is likely the most problematic methodological flaw that eliminates the statistical significance of the difference in the response rate between the active and placebo groups (Kirsch et al., 2008; Fournier et al., 2010).

Another important methodological consideration is that even if the rating scales (such as the HDRS) on which the treatment response is assessed are robust and reliable, not all items of the scale (e.g., anxiety, somatic, early insomnia, hypochondriasis, and somatic symptoms) represent equal proportions of the observed change in the global score (Nelson et al., 2005). This could explain why a high level of anxiety symptoms could result in an underestimation of the response to treatment and why anxiety disorders are frequently associated with TRD (Souery et al., 2007). Other clinical characteristics are associated with a higher risk of low response to ADs. In particular, some MDD patients who meet the criteria for TRD are later revealed to suffer from bipolar disorder (Fekadu et al., 2012). Bipolar depression is less prone to respond to ADs than MDD (Gijsman et al., 2004), even though recent findings suggest that ADs may be as effective against bipolar depression as against MDD (Tondo et al., 2013; Vázquez et al., 2013).

Among clinical characteristics, older age has also been shown to be associated with a lower response rate to ADs. In a meta-analysis conducted on 15 late life MMD trials and 59 adult MDD trials, it was found that the response rate drops from 53.9% in adults to 45.2% in older patients (Tedeschini et al., 2011). In adolescents, response rate has been shown to be much higher, several studies reporting response rate at week 36 from 65 (Tao et al., 2009) to 80% (March et al., 2006), with a mean remission rate of 67% (Cox et al., 2012). Whether age itself may explain the difference remains unclear because somatic comorbidities may have a role in increasing the risk of non-response or partial response to ADs in older patients, particularly cardiac (Scherrer et al., 2012), cerebrovascular (Miller et al., 2002), and neurodegenerative disorders (Price et al., 2011).

Sex also constitutes a clinical characteristic associated with difference in AD treatment response. Indeed, a study suggested that men respond more favorably to imipramine than women, and premenopausal women more frequently to fluvoxamine than men (Vermeiden et al., 2010). In animal studies, Goel et al. (2011) showed that acute citalopram induced higher neuronal activation in male brain than in females or gonadectomized males. This suggests a gonadal hormone influence on complex interactions between serotonin and neural circuits that mediate the stress axis (see section HPA Axis Regulation below) and could therefore explain some of the sex differences in the response to AD.

The lack of response to ADs may also be the consequence of non-adherence to the treatment as the rate of adherence to ADs has been estimated to be particularly low, varying over a period of six months from 12.4% for patients taking older MAOIs and TCAs to 29.3% for those taking SSRIs and 33.6% for those taking SNRIs (Sheehan et al., 2008). In a 9-week follow-up period, up to 20% of patients missed taking their treatment for at least four consecutive days (Demyttenaere et al., 2001). This poor compliance has been shown to alter the estimation of response and remission rates (Akerblad et al., 2006). According to Jin et al. (2008), various factors contribute to non-compliance. Jeon-Slaughter (2012) found that low income level, combined with health insurance status and race/ethnicity, predicted non-adherence to ADs. This was confirmed in a recent review (Rivero-Santana et al., 2013) that demonstrated that younger people were less compliant than older patients and minority ethnic patients were less compliant than white patients. Non-pharmacological interventions can improve the adherence to AD treatments. Vergouwen et al. (2003) found that collaborative interventions in primary care were associated with clinical benefit, particularly in patients suffering from MDD who were prescribed adequate dosages of ADs.

## Predictors of poor response to antidepressant therapy: pharmacological component

### Drug metabolism

Availability of the drug to its brain targets is one of the first requisite conditions of its effect and clinical impact. However, various conditions affect drug delivery such as its metabolism. Drug metabolism is altered by a wide range of factors such as age, rate of expression of drug metabolism systems, comorbid disease, sex, pregnancy, environment, drug dose, enzyme induction/inhibition, diet, genetics… For instance, younger people metabolize drugs faster than elderly people, men faster than women, and smokers faster than non-smokers. AD medications are metabolized mainly in the liver into compounds that are typically pharmacologically active but with different properties than the parent drug. Drug metabolism may result in poor response. Hepatic metabolism mainly occurs via cytochrome P450 (CYP) enzymes, which comprise more than 200 isoenzymes (mainly CYP1A2, CYP2B6, CYP2D6, CYP2C9, CYP2C19, and CYP3A4/5): they account for 75% of drug metabolism (Guengerich, 2008), particularly oxidative metabolism.

Variations in CYP genes have been shown to be associated with modified pharmacokinetic clearance of ADs. In humans, CYP is encoded by 18 families and 43 subfamilies of genes, corresponding to 57 genes and more than 59 pseudogenes. Thus, many different genes may alter CYP, some patients being poor and others ultrarapid metabolizers. When ultrarapid metabolizers are treated with typical doses of ADs, they have low plasma concentrations and do not respond. Polymorphisms in genes for crucial CYP enzymes, such as CYP2D6 or CYP2C19, alter the metabolism of ADs and thus their plasma concentrations (Brosen, 2004). Carriers of the non-functional allele of CYP2C19 exhibit a 42% decrease in clearance of the SSRI citalopram compared to carriers of the functional allele (Yin et al., 2006). Bondolfi et al. (1996) investigated seven non-responders to citalopram; six were extensive CYP2D6 metabolizers, and all seven were extensive CYP2C19 metabolizers. Furthermore, when administered an inhibitor of these two enzymes, citalopram serum levels rose in all subjects, as well as the therapeutic response. However, Grasmäder et al. (2004) found that plasma concentrations of several ADs were altered depending on the CYP2D6 and CYP2C19 genotype, even if this genotype was unrelated to clinical response. Peters et al. (2008) using subjects from the STAR^*^D study found no association between 15 polymorphisms of four P450 genes (CYP2D6, CYP2C19, CYP3A4, and CYP3A5) and citalopram response. More recently, Mrazek et al. (2011) examined data from the white non-Hispanic subjects who were treated with citalopram in the same STAR^*^D sample. They found a modest association between CYP2C19 variation and remission following citalopram, particularly in a subset of patients able to tolerate the medication. Thus, evidence on the association between genetic variations in CYP and AD response is inconsistent and likely depends on the patients and drug used.

Among the environmental factors that influence pharmacokinetics, smoking, treatment adherence, and concurrent medications are particularly important. There are numerous drug interactions with cigarette smoking. Suzuki et al. (2011) found that smoking status significantly affected fluvoxamine concentration (only in the low 50 mg/d dose group). Together, CYP2D6 genotype and smoking status explained 23% of the variance in fluvoxamine concentration in this group.

Failure to respond to or tolerate a drug may be related to comorbid medical conditions (hepatic or renal insufficiency, cardiovascular disease) and/or to its related polypharmacy. Comorbid medical conditions that alter hepatic function are likely to decrease the rate of drug metabolism. In addition, co-prescriptions increase the risk of drug-drug interactions with ADs in the treatment of comorbid illness. Drug interactions are more likely to occur with high-risk drugs, such as fluvoxamine, fluoxetine, paroxetine, and nefazodone (Richelson, 1998). Coelho and Brum Cde (2009) investigated the interactions between ADs and antihypertensive and glucose-lowering drugs at two primary care units and found that 19 of 29 patients were exposed to 47 interactions involving pharmacokinetic and pharmacodynamic mechanisms. When initiating a new prescription, the physician should select an AD while considering comorbid medical conditions, including dosage adjustment, possible drug interactions, adverse effects, and tolerability issues. The physician should also inform the patient about the influence of co-administered drugs and simultaneous intake of beverages and food on the bioavailability of drugs. For instance, grapefruit juice consumption increases the mean peak plasma concentrations and the concentration-time curve of sertraline (Ueda et al., 2009) and fluvoxamine (Hori et al., 2003).

In case of TRD, therapeutic drug monitoring is a valuable tool for tailoring the dosage of the prescribed medication to the individual characteristics. Dose titration is strongly recommended to achieve therapeutic plasma concentrations that allow for the highest probability of response or remission. In addition to drug concentration measurements, symptom rating by the treating physician at baseline and at week 2 is recommended (Hiemke et al., 2011). In certain situations, AD monitoring could be combined with pharmacogenetic metabolism tests (Hiemke et al., 2011). For instance, when the concentrations are outside the reference range, pharmacogenetic tests could be recommended to detect polymorphisms that give rise to slow/rapid metabolizers. Winner et al. (2013) recently demonstrated that pharmacogenomic-directed prescribing reduced the incidence of adverse drug reactions and improved the efficacy of AD medication regimens. Thus, pharmacogenomic testing to determine metabolic capacity may be a valuable strategy to recognize individuals who will obtain a therapeutic benefit from a drug.

### Blood-brain barrier

Among the mechanisms of poor response to ADs, the drug efflux transporters that are expressed at the blood-brain barrier (BBB) and enable drugs to access the brain play a major role. The BBB is composed of brain capillary endothelial cells in association with pericytes and smooth muscle cells that delineate the circulating blood from glial cells and the neuronal terminals of the central nervous system. The BBB limits the traffic of substances to trans-cellular transport rather through the intercellular spaces because the tight junction system of the trans-membrane proteins acts as a physical barrier. Consequently, only lipophilic compounds of low molecular weight are able to cross the BBB. However, various transport systems ensure wider exchanges through the BBB, including the ATP-binding cassette (ABC transporters) system (for lipid-soluble molecules) (Benarroch, 2012). The human genome encodes 49 different ABC transporter proteins classified into seven subfamilies (ABCA to ABCG) (Dean et al., 2001). The P-glycoprotein (P-gp) encoded by the multi-drug resistance 1 (MDR1/ABCB1) gene, the breast cancer resistance protein encoded by the ABCG2 gene, and the multidrug resistance-associated proteins 4 and 5 are expressed by the brain endothelial cells and ensure active efflux of lipid-soluble molecules from the brain, reducing penetration of drugs into the brain. Compounds that interact with ABC transporters can be classified as substrates, modulators, or inhibitors. AD drugs interact mainly with P-gp. Thus, ADs that are substrates of P-gp are subject to greater efflux from brain endothelial cells and decreased penetration into the brain. Moreover, drug-drug interactions can also be the consequence of competing/synergic effects on P-gp. Several drugs, including cyclosporine, nifedipine, quinidine, and verapamil, are P-gp inhibitors (O'Brien et al., 2012). *In vivo* preclinical studies, particularly in P-gp knockout mice, have demonstrated that not all ADs are subject to the same level of limitation to brain penetration by P-gp (Uhr et al., 2000, 2003; Uhr and Grauer, 2003; Karlsson et al., 2013). Moreover, metabolites of some ADs may not be substrates of P-gp, in contrast to their parent molecules (Weiss et al., 2003; Grauer and Uhr, 2004; Wang et al., 2008a). Clinical evidence of the role of P-gp in the response to ADs has been provided by studies of variants of the ABCB1 gene. Several single nucleotide polymorphisms (SNPs) of the ABCB1 gene have been identified and associated with a decreased clinical response to AD (Kato et al., 2008; Uhr et al., 2008; Sarginson et al., 2010; Lin et al., 2011; Singh et al., 2012) as well as a poorer tolerance profile (Roberts et al., 2002; Jensen et al., 2012; de Klerk et al., 2012), although several studies failed to replicate these results (Laika et al., 2006; Mihaljevic Peles et al., 2008; Menu et al., 2010). Furthermore, endogenous and synthetic glucocorticoids also act as P-gp substrates (Ueda et al., 1992; Schinkel et al., 1995; Uhr et al., 2002). Hyperactivity of the hypothalamus-pituitary-adrenal (HPA) axis is one of the most consistent biological hallmarks of MDD, and it has been suggested that increased penetration of glucocorticoids into the brain as a result of P-gp inhibition may contribute to normalization of HPA axis hyperactivity in MDD (O'Brien et al., 2012). These data suggest evaluation of P-gp inhibition as an augmentation strategy for improving response to AD therapy.

## Predictors of poor response to antidepressant therapy: neurobiological components

Based on the understanding we have of the neurobiological mechanisms of action of ADs, the response to ADs can be explored at the following levels: brain structures, neurotransmission, and molecular targets. We will now describe each of these mechanisms (Table 1).

### Brain structures and response to antidepressants

Various studies have explored brain changes associated with response to ADs by using electroencephalography (EEG) (alpha and theta activities) or neuroimaging (Functional magnetic resonance imaging: fMRI, Positron emission tomography: PET) that allow deducing potential mechanisms and markers of response to ADs.

Brain activity measurements by quantitative EEG in the resting state or during simple tasks have been used to predict response to ADs. Ulrich et al. (1986) observed increased alpha rhythmic activity (8–12 Hz) in the posterior regions of the head on both sides that was higher in amplitude on the dominant side in patients responding to amitriptyline. Subsequently, Knott et al. (1996) observed higher alpha and less theta rhythmic activity (4–7 Hz) among imipramine-responders than non-responders. Bruder et al. (2001) observed a difference in alpha asymmetry between fluoxetine responders and non-responders; non-responders displayed reduced alpha activity over the left hemisphere than the right, whereas responders tended to have the opposite asymmetry. Other studies focused on the brain regions associated with this altered alpha activity. Bruder et al. (2008) demonstrated that the difference between SSRI responders and non-responders involved occipital areas, where differences in alpha asymmetry were also observed. Theta activity was also investigated. EEG theta frequencies are generated in various brain areas, such as the medial prefrontal cortex (PFC), anterior cingulate cortex (ACC), hippocampus, amygdala, and ventral striatum. In the ACC, Pizzagalli et al. (2001) found an association between pre-treatment theta increases in rostral ACC and responses to nortriptyline. Mulert et al. (2007) reported similar findings with citalopram or reboxetine. This pre-treatment change in theta power in relationship to AD outcome has not been consistently observed (Cook et al., 2002). However, they demonstrated that the decrease in prefrontal cordance (i.e., the measure of quantitative EEG power that characterizes PFC function) that occurs after 1 week of treatment only in responders is also predictive of a better final outcome (Cook et al., 2002, 2009). In another study, Bares et al. (2008) found that reduction in the PFC theta quantitative EEG cordance value after the first week of treatment can predict the response to venlafaxine. Quantitative EEG measurements are now considered a promising clinical tool for predicting conventional AD treatment response (Leuchter et al., 2009, 2010). Interestingly, brain electrical activity has also been used to predict outcomes of non-conventional ADs, such as the NMDA receptor antagonist ketamine or non-pharmacological AD therapy, such as deep brain stimulation (DBS). For example, Duncan et al. (2013) recently demonstrated that measuring sleep slow wave activity (0.6–4 Hz) could predict ketamine response in individuals with TRD, whereas frontal theta quantitative EEG cordance has been shown to predict long-term AD response to subcallosal cingulate DBS in TRD patients (Broadway et al., 2012).

The involvement of the rostral ACC in TRD or treatment non-response is also supported by neuroimaging studies. Indeed, PET studies have observed increased baseline rostral ACC activity in MDD patients who subsequently responded to ADs (Mayberg et al., 1997; Saxena et al., 2003) while an fMRI study demonstrated that higher ACC activity during precise tasks, such as processing negative stimuli, was associated with the most robust treatment response (Davidson et al., 2003). Moreover, ACC activation during unsuccessful motor inhibition predicted response to escitalopram (Langenecker et al., 2007). Interestingly, a recent study showed that increased activity in the rostral ACC is a predictor not only of treatment outcome to conventional ADs but also to putative ADs, such as ketamine (Salvadore et al., 2009), or to non-pharmacological therapy, such as sleep deprivation (Wu et al., 1999). Non-response to ADs is correlated with low pre-treatment activity in the rostral ACC (Mayberg et al., 1997; Pizzagalli, 2011), which is one of the most reliable markers for predicting treatment outcome for conventional monoaminergic-acting ADs, intravenous ketamine treatment, and non-pharmacological treatments, such as electroconvulsive therapy (ECT) or repetitive transcranial magnetic stimulation (rTMS) (Pizzagalli, 2011). However, a low level of activity in the rostral ACC predicts a better outcome for cognitive behavioral therapy (CBT) (Fu et al., 2008; Pizzagalli, 2011; Roiser et al., 2012).

Gray matter brain volumes and, more recently, forebrain white matter integrity have also been measured in patients with MDD (first episode, remittent, or TRD). Frontolimbic gray matter areas (medial and orbital PFC) were reduced in the most severely depressed individuals (i.e., treatment-resistant/chronic group) (Serra-Blasco et al., 2013). Moreover, patients that did not respond to escitalopram exhibited microstructural abnormalities in fiber tracts connecting the cortex with limbic structures, such as the ACC (Alexopoulos et al., 2008). In addition, patients with TRD displayed abnormalities of both internal and external capsule integrity connecting cortical to subcortical nuclei and of the corpus callosum (Guo et al., 2012). Interestingly, these corticolimbic pathways are negatively impacted by adverse life events or by genetic polymorphism of the serotonin transporter (Alexopoulos et al., 2009; Choi et al., 2009), and corticolimbic connectivity increased as scores on the HDRS decreased during treatment (Anand et al., 2005). This suggests that assessing corticolimbic connectivity could be used to predict AD outcome. Further, the PFC region is one of the favorite targets for DBS in patients with TRD (Mayberg et al., 2005; Lozano et al., 2008; Malone et al., 2009). In addition, Dunkin et al. (2000) reported pre-treatment difference between fluoxetine responders and non-responders on PFC-related tasks reflecting executive dysfunction. Recently, Gupta et al. (2013) demonstrated that patients with TRD exhibited mildly reduced performance across all neurocognitive domains with a superimposed moderate impairment in verbal working memory. Finally, Bruder et al. (2004) investigated dichotic listening and demonstrated that patients who respond to SSRIs differed from non-responders in favoring left- over right-hemisphere processing of dichotic stimuli. Fluoxetine responders displayed greater left-hemisphere advantage for words and less right-hemisphere advantage for complex tones compared to non-responders. The cognitive, sensorial, or behavioral alterations shown in AD responders vs. non-responders are likely related to differences in the functioning of the brain areas underlying these functions, and thus, this suggests that brain alterations may also be valuable predictors of AD outcome.

The ACC is not the sole brain region whose activity can predict AD response. Indeed, improvement of MDD symptoms after AD has also been associated with lower pre-treatment metabolism (detected by PET) in the amygdala and thalamus and with higher pre-treatment metabolism in the medial PFC (Saxena et al., 2003). The hippocampus has also received some interest, and a recent study showed that MDD patients who met criteria for clinical remission at 8 weeks of AD treatment had larger pre-treatment hippocampal volumes than non-remitters, suggesting involvement of the hippocampus not only in the pathophysiology of MDD but also in treatment outcome (MacQueen et al., 2008; McKinnon et al., 2009). Larger hippocampal volume was also associated with a lower probability of relapse (Kronmüller et al., 2008). Microstructural abnormalities in the hippocampus have been suggested to indicate the vulnerability to treatment resistance (Ruhé et al., 2012). Further, a recent study pointed to a crucial role of the right insula: indeed, insula hypometabolism (detected via PET) was associated with poor response to escitalopram while the opposite was observed concerning cognitive behavior therapy, as insula hypometabolism was associated with good response to this cognitive therapeutic approach (McGrath et al., 2013). This indicates that neuroimaging can also help in selecting the appropriate treatment, and that brain activity does not predict poor response to treatment in a general way, but poor response to a particular therapeutic approach.

The crucial role of corticolimbic areas in the response to AD therapy has also been highlighted by recent impressive preclinical studies. These studies highlight the mechanisms underlying AD action in a given region. For example, Vialou et al. (2010) demonstrated that DeltaFosB induction in the nucleus accumbens was required for the effects of fluoxetine in the social defeat test, whereas Li et al. (2010) demonstrated that the effects of ketamine require synapse formation (particularly mTor-dependent synapse formation) in the PFC. Further, a recent paper showed that neural activity in the visual cortex during emotional processing predicts the response to scopolamine in depression (Furey et al., 2013).

Finally, TRD and/or treatment non-response can also be indirectly approached by studying the mechanism of non-pharmacological treatments, including vagus nerve stimulation therapy (VNS), ECT, and DBS. VNS is approved for the treatment of TRD. In VNS, a battery-powered generator is implanted in the chest wall and connected to a wire wrapped around the left vagus nerve in the neck. This wire sends intermittent electrical pulses through the vagal afferent connections to the brainstem, which may alter information processing in brain regions to which it projects, including the noradrenergic locus cœruleus and serotonergic nuclei as well as the thalamus, hypothalamus, central amygdala nucleus, bed nucleus of the stria terminalis, and nucleus accumbens, which are all disrupted during MDD. Functional imaging suggests that VNS leads to activity changes in the hypothalamus, orbitofrontal cortex, amygdala, hippocampus, insula, medial PFC, and cingulate (Bohning et al., 2001; Zobel et al., 2005), suggesting that VNS might aid in the recovery of MDD patients by reversing the pathophysiological alterations observed in MDD. ECT is based on the administration of brief electrical pulses to the scalp to induce depolarization of cortical neurons and thus brain seizures. It is among the most effective treatments for TRD and AD non-response. The mechanism of action of ECT remains a mystery, but the recent observation that electroconvulsive shock, the animal analogue of ECT, stimulates precursor cell proliferation in the subgranular zone of the dentate gyrus as well as hippocampal neurogenesis in the rat (Madsen et al., 2000) and monkey (Perera et al., 2007) suggests that pharmacological treatments and ECT may target a common endpoint, such as neuronal plasticity. Finally, because a number of clinical studies have demonstrated long-term effects of DBS in terms of improving symptoms of MDD (Bewernick et al., 2012; Lozano et al., 2012), the study of the neurobiological mechanisms underlying the beneficial effects of DBS will contribute to the understanding of TRD and of the mechanisms underlying poor response to AD. For example, a recent study by Schmuckermair et al. (2013) in a mouse model of TRD demonstrated that repeated nucleus accumbens DBS reversed depression-related behavior and coincided with changes in stress-induced neuronal activation of prelimbic, infralimbic, and cingulate areas in the lateral habenula and in the dentate gyrus of the hippocampus, where neurogenesis was also increased. In addition, Hamani et al. (2010) demonstrated that DBS in the rat ventromedial PFC induced a clear AD-like effect that was dependent on the integrity of the serotoninergic system.

### Neurotransmission and response to antidepressants

#### Serotoninergic system

Because TCAs and SSRIs increase serotonin (5-HT: 5-hydroxytriptamine) availability in the synaptic cleft, the level of involvement of 5-HT in predicting AD response has been a focus of research. Consistent with predictions, tryptophan depletion (which is a precursor of the 5-HT synthesis) in subjects successfully treated with SSRI prevents AD effects (Delgado et al., 1999), indicating that 5-HT is essential for the action of SSRIs. More recently, the utilization of PET permitted the precise localization of this 5-HT action. SSRI treatment outcome was related to serotonin transporter (SERT) ratios between the raphe nuclei and serotonergic projection areas (habenula, amygdala, hippocampus, and subgenual cingulate cortex) before treatment (Lanzenberger et al., 2012). In animal models, depletion of 5-HT tissue content by para-chlorophenylalanine, an inhibitor of tryptophan hydroxylase (TPH), prevented the acute effects of SSRIs in a bioassay (O'Leary et al., 2007). Rodent studies also permitted the determination of the precise molecular target of AD action, particularly through the use of knockout mice for SERT and several 5-HT receptors. As expected, in a bioassay assessing effects of sub-acute injections of AD, the action of fluoxetine was abolished in SERT knockout mice, whereas the effect of a noradrenaline-preferring AD, desipramine, was conserved (Holmes et al., 2002). Once the blockade of the SERT has been achieved, the increased 5-HT in the synaptic cleft will bind to several 5-HT receptors. One of the most studied is the 5-HT_1A_ postsynaptic receptor. In initial studies, SSRIs failed to alter immobility in 5-HT_1A_ mutant mice, suggesting that 5HT_1A_ receptors are critical for the expression of AD-like responses to SSRIs (Mayorga et al., 2001; Santarelli et al., 2003). However, a more complex picture later emerged as other studies showed that the effects of SSRIs were still present in 5-HT_1A_ mutant mice (Guilloux et al., 2006; Holick et al., 2008). In fact, the use of 5-HT_1A_ mutants did not allow to distinguish the pre- and the post-synaptic 5-HT_1A_ receptors, and it is probable that the involvement of these receptors during chronic SSRIs is dynamic, as in the initial phase of the treatment the action of 5-HT on the 5-HT_1A_ somatodendritic receptors may oppose the action on the post-synaptic receptors, while during the second phase, the presynaptic receptors get desensitized (see below). It has also been shown that desipramine, a NRI, still exerts an AD-like effect in 5HT_1A_ receptor knockout mice despite reduced baseline immobility in the tail suspension test (Mayorga et al., 2001). However, the action on the postsynaptic 5-HT_1A_ receptors is compromised by a concomitant action on 5-HT_1A_ autoreceptors located in the raphe, which reduces the clinical efficacy of SSRIs and partly explains their delayed onset of action. Using conditional knockout mice for pre-synaptic 5-HT_1A_ receptors, it was observed that reduction of 5-HT_1A_-autoreceptor expression with unchanged post-synaptic 5-HT_1A_ receptor expression induced AD-like behavior and augmented SSRI effects (Richardson-Jones et al., 2010). These observations were recently confirmed using another experimental strategy, intra-raphe infusion of small-interfering RNA (siRNA) sequences directed toward the 5-HT_1A_ autoreceptors. The siRNA decreased the expression of these receptors without affecting post-synaptic 5-HT_1A_ receptors, concomitant with a robust and rapid AD-like effect (Bortolozzi, Castañé, Semakova, Santana, Alvarado and Cortés, 2012).

The involvement of 5-HT_1B_ receptors is complex and depends upon the class of AD used, as well as on the methodology used to study their contribution (knockouts or pharmacological studies). Indeed, in 5HT_1B_ receptor knockout mice, desipramine still has AD-like effects (Mayorga et al., 2001) while pharmacological blockade of the 5-HT_1B_ receptors with GR 127935 or SB216641 (5-HT_1B_ receptor antagonists) potentiated the effects of the drug (Tatarczyñska et al., 2004). Concerning SSRIs, an increased sensitivity to these compounds has been observed in mutants (single mutants for the 5-HT_1B_ receptors) or after 5-HT_1B_ receptors antagonists (Mayorga et al., 2001), whereas other studies reported SSRI resistance in the mutants (Trillat et al., 1998). Finally, in double knockout for 5-HT_1A_ and the 5-HT_1B_ receptors the response to acute SSRI was impacted, but not the one to chronic SSRIs (Guilloux et al., 2011). Other 5-HT receptors are required for AD action, including the 5-HT_2B_ (Diaz et al., 2012), 5-HT_2C_ (Cryan and Lucki, 2000a) and 5-HT_4_ receptors (Cryan and Lucki, 2000b; Lucas et al., 2007).

Genetic studies confirmed the involvement of 5-HT-related targets in the outcome of 5-HT therapy. For example, different studies indicated a polymorphism in the human gene encoding SERT (*SLC6A4*) as a predictor of response to AD. Heils et al. (1996) identified a functional polymorphism in the transcriptional region upstream of the *SLC6A4*-coding sequence (5-HTTLPR) that affects *SLC6A4* expression, in which the *l* allele yields twofold higher *SLC6A4* expression in the basal state than the *s* form. In a meta-analysis of the literature, Kato and Serretti (2010) demonstrated that the *l* variant is associated with a better response rate to AD than the *s* allele. Nakamura et al. (2000) further examined the polymorphic region and concluded that the alleles previously reported as *s* and *l* should be respectively divided into four (14A, 14B, 14C, and 14D) and six allelic variants (16A–16F). Smeraldi et al. (2006) demonstrated that among carriers of the *l* variant, 16F *l* carriers exhibited only a partial response to AD and 16D *l* carriers exhibited a marginally better response than 16A *l* allele carriers. Another polymorphism of *SLC6A4* has been associated with an increased response to ADs; carriers with the 12 allele displayed a greater response to ADs, particularly when this allele was also associated with the *l* variant of the SERT-linked polymorphic region (*5-HTTLPR*) gene (see (Kato and Serretti, 2010) for a review). However, these findings have not been consistently replicated.

Serotonin biosynthesis involves TPH. This enzyme has two isoforms encoded by TPH1 and TPH2 genes, and SNPs of both genes have been reported. A significant association of TPH1 218A/C with response to ADs has been reported (Serretti et al., 2001) but these findings were not replicated (Kato et al., 2007).

A total of 50 SNPs of the 5-HT_1A_ receptor gene have been described. Of particular interest is the 1019C/G (rs6295) SNP, which is related to the altered expression and function of 5-HT_1A_ receptors. Indeed, the G allele is associated with an increase in 5-HT_1A_ autoreceptors and thus a decrease in 5-HT neurotransmission. An association of this polymorphism with AD response was found only in Asian and not in Caucasian populations (Kato and Serretti, 2010). Two important common SNPs of the gene encoding the 5-HT_2A_ receptor, 102T/C and 1438A/G, have been described: the C variant of the 102T/C SNP is associated with lower 5-HT_2A_ receptor expression compared to the T variant, whereas the A variant of the 1438A/G SNP increases promoter activity compared to the G variant. Interestingly, the AD response was higher among the G/G genotype carriers than among the A/G or A/A carriers, although only in the Asian population (Kato and Serretti, 2010).

Polymorphisms combine with environmental factors in the etiology of MDD (El-Hage et al., 2009). For example, stressful life events predict a better response to escitalopram, but polymorphisms in 5-HT related genes, such as *5-HTTLPR*, alter these effects (Keers et al., 2011). Assessment of 5-HT-related polymorphisms or pre-treatment PET of 5-HT molecular targets would improve the prediction of treatment response. Specifically, targeting 5-HT_1A_ autoreceptors to eliminate their initial negative contribution during AD therapy would accelerate the onset of the beneficial effects of AD therapy.

#### Noradrenergic system

Drugs such as the TCA desipramine, and other TCAs that increase noradrenaline and serotonine neurotransmission, as well as the NRI reboxetine act by binding to the noradrenaline transporter which increases noradrenaline levels in the synaptic cleft, activating noradrenergic receptors. The enzyme dopamine beta-hydroxylase (*Dbh*) is responsible for the synthesis of epinephrine and noradrenaline. Mice unable to synthesize noradrenaline and epinephrine due to targeted disruption of the *Dbh* gene did not exhibit altered behavior in bioassays for depressive-like behavior, but ADs with a noradrenergic-preferring action such as desipramine or reboxetine failed to exert AD-like effects (Cryan et al., 2001). The same results were obtained with the MAOI pargyline and the atypical AD bupropion (Cryan et al., 2004). Surprisingly, the effects of the SSRIs fluoxetine, sertraline, and paroxetine were also absent or severely attenuated in *Dbh* knockout mice while the effects of another SSRI, citalopram, were not altered. Restoration of a normal noradrenergic level by L-*threo*-3,4-dihydroxyphenylserine restored the behavioral effects of both desipramine and paroxetine in the knockout mice, demonstrating that the AD non-response was due to altered noradrenergic function rather than developmental abnormalities resulting from chronic noradrenaline deficiency. Thus, noradrenaline may be involved in the effects of not only noradrenaline-acting AD drugs but also 5-HT-acting compounds. The beneficial action of noradrenergic-acting compounds in the treatment of MDD may be related to the α_2_-adrenergic receptor because the AD-like effects of desipramine are reversed by α_2_-adrenergic receptor antagonists, such as yohimbine or idazoxan (Yalcin et al., 2005; Zhang et al., 2009). Other adrenergic receptors are also involved: the cognitive effects of these treatments are mediated by post-synaptic α_1_-adrenergic receptors in the mPFC (Bondi et al., 2010). However, β_2_ or β_3_ adrenergic receptors do not appear to play a pivotal role in AD effects (Zhang et al., 2009; Stemmelin et al., 2010).

Finally, the involvement of organic cation transporter 2 (OCT2) in non-response to AD is also of note. OCT2 is involved in monoamine clearance, and mice deficient in this protein exhibited an altered response to AD (Bacq et al., 2012).

The contribution of the noradrenergic system to AD effects is largely confirmed by genetic data. Several polymorphisms of the gene encoding the noradrenalin transporter (*SLC6A4*) have been associated with AD response, particularly the rs2242466 (–182T/C) and rs5569 (1287G/A) polymorphisms (Shiroma et al., 2010). Catechol-*O*-methyltransferase (COMT) plays a pivotal role in the degradation of noradrenalin and dopamine. Interestingly, the Val158Met (rs4680) polymorphism of the *COMT* gene is associated with AD response (Benedetti et al., 2009; Tsai et al., 2009). Baune et al. (2008) demonstrated a negative influence of the higher activity COMT 158Val/Val genotype on AD response during the first 6 weeks of treatment, possibly due to the consequent decrease in dopamine availability. MAOA is involved in the degradation of monoamines, and polymorphisms of MAOA have been associated with fluoxetine, paroxetine, or mirtazapine response (Yu et al., 2005; Tadić et al., 2007; Domschke et al., 2008b).

Xu et al. (2011) found that early life stress may interact with the *SLC6A2* polymorphism to alter AD response. Such effects might occur via epigenetic mechanisms, such as methylation or acetylation.

The assessment of polymorphisms of noradrenergic-related genes would improve the prediction of AD response. Combining ADs with α_2_-adrenergic receptor agonists could also improve the response rate or accelerate the onset of therapeutic action.

#### Other neurotransmission systems

One reason for non-response to ADs targeting monoaminergic neurotransmission is that these drugs may be ineffective in patients with alterations of other neurotransmission systems. For example, psychomotor retardation, a symptom exhibited by a subgroup of patients with MDD, has been related to a deficit in dopaminergic neurotransmission, particularly in the dorsolateral PFC. Consequently, these patients may preferentially benefit from treatment directly targeting dopaminergic neurotransmission. This has been explored by Taylor et al. (2006), who demonstrated that MDD patients with reduced pre-treatment performance on neuropsychological tests had a poor outcome after 12 weeks of fluoxetine treatment.

Substance P has also been suggested to have a key role in AD response. For example, decreased substance P in the cerebrospinal fluid has been associated with poor response to ADs (Carpenter et al., 2008). This conclusion is supported by convincing genetic data because the D allele of the angiotensin-converting enzyme (ACE) gene, which is related to higher ACE plasma levels, is associated with higher substance P levels and a more rapid onset of AD response (Baghai et al., 2004; Bondy et al., 2005; Narasimhan and Lohoff, 2012).

There is increasing evidence for the involvement of glutamate neurotransmission in MDD, and glutamate receptors are now being explored as targets for the treatment of MDD. For example, a clinical effect was observed when riluzole, a glutamatergic-acting compound, was added to ongoing AD therapy in TRD patients (Sanacora et al., 2007, 2008). Ketamine has a rapid AD effect and improves symptoms in TRD patients or in patients not responding to ECT (Ibrahim et al., 2011).

This is confirmed by genetic studies. For example, studies have found an association between a SNP of the dystrobrevin-binding-protein 1 gene, which is involved in glutamatergic neurotransmission, and AD response (Pae et al., 2007b; Kim et al., 2008). Furthermore, according to the STAR^*^D study, a SNP (rs1954787) of the *GRIK4* (glutamate receptor ionotropic kainate 4) gene encoding kainate receptor subunit 1 is associated with response to citalopram (Mayer, 2007; Horstmann and Binder, 2009; Stawski et al., 2010; Narasimhan and Lohoff, 2012). This was confirmed in another cohort, the “Munich Antidepressant Response Signature” (MARS) project (Horstmann et al., 2008, 2010; Porcelli et al., 2011). Horstmann et al. (2010) identified another SNP (rs12800734) in the *GRIK4* gene that is more strongly associated with response to treatment.

Data to support the involvement of GABA (gamma-aminobutyric acid) in the response to ADs are sparse, and this neurotransmitter does not appear to have a pivotal role in AD effects. However, mice with a deletion of the gene encoding the GABA transporter subtype 1 (*GAT1*), which transports extracellular GABA into presynaptic neurons, exhibited non-response to fluoxetine and amitriptyline (Liu et al., 2007a).

The endocannabinoid system is a modulatory system with both central and peripheral actions. Two cannabinoid receptors have been characterized: CNR1 located predominantly in the brain, and CNR2 in peripheral immune tissue and in glial cells in the central nervous system. Interestingly, knockout mice for the CNR1 receptor displayed attenuated response to desipramine and paroxetine (Steiner et al., 2008). Mitjans et al. (2012, 2013) demonstrated that genetic variability in endocannabinoid receptors could play a role in clinical response. Specifically, molecular variations in the *CNR1* gene appear to differentiate the response to citalopram according to sex. In an analysis of SNP variability in the *CNR1* gene, Domschke et al. (2008a) reported that the G allele of rs1049353 leads to increased risk of non-response to in female patients. These results suggest a role of the *CNR1* gene in the etiology of MDD and clinical response to citalopram.

Leptin signaling may be involved in the pathophysiology of MDD. Kloiber et al. (2013) recently suggested an association of polymorphisms in the leptin gene with failure of AD to achieve remission. In this study, decreased leptin serum levels and reduced leptin mRNA expression were detected in patients with impaired treatment response, independently of their genotype configuration.

Endogenous opioids are involved in the regulation of mood and behavior. Three receptors (mu, delta, and kappa) interact with a family of endogenous opioid peptides (β-endorphin, enkephalins, and dynorphins). Studies in a mouse model of MDD have demonstrated that the combination of monoaminergic ADs and opioid receptor agonists can produce synergistic AD effects (Berrocoso and Mico, 2009). The mu receptor has been associated with citalopram response (Garriock et al., 2010). Haj-Mirzaian et al. (2013) demonstrated that elevated levels of endogenous opioids and nitric oxide due to bile-duct ligation in mice induced an AD-like effect. The effect was reversed by blockade of the nitrergic and opioid systems, suggesting an involvement of these systems in non-response. However, it is difficult to evaluate the risk-benefit balance of currently available mu opioid receptors agonists as ADs, partly because of their inherent abuse liability.

### Neural plasticity and response to antidepressants

#### Molecular aspects

Once the AD has increased monoamines in the synaptic cleft or bound to post-synaptic serotoninergic or noradrenergic receptors, it activates second messengers, such as the cyclic adenosine monophosphate (cAMP) pathway, leading to the production of cAMP-dependent protein kinase (PKA). This activation may in turn stimulate nuclear transcription factors, such as cAMP response element binding protein (CREB), via phosphorylation. Activated CREB enhances the transcription of many target genes, including brain-derived neurotrophic factor (BDNF), which exerts its effects mainly by binding to its specific receptor: the tyrosine receptor kinase B (TrkB). Consequently, non-response to AD has been investigated in relationship to alterations of these targets, particularly when polymorphisms of the genes encoding these proteins have been reported.

BDNF secretion and intercellular trafficking are related to a SNP in the *BDNF* gene that causes a valine to methionine substitution (Val66Met). A meta-analysis by Kato and Serretti (2010) indicated a better response to ADs in Met allele carriers. Other neurotrophic/growth factors have also been implicated in TRD and/or in AD response, including vascular endothelial growth factor (VEGF), fibroblast growth factor 2, and insulin-like growth factor 1 (IGF-1).

In CREB mutant mice (CREBaD), the SSRI fluoxetine and NRI desipramine still induce AD-like effect in bioassays for depressive behavior (Conti et al., 2002). This effect is accompanied by a desipramine-induced attenuation of the stress-induced activation of the HPA axis in both CREBaD-deficient and control mice (Conti et al., 2002). Interestingly, the AD-induced increase in BDNF in the cortex and hippocampus is absent in CREBaD-deficient mice (Conti et al., 2002), indicating that CREB may be a critical mediator of the transcriptional effects of AD.

Heterozygous *bdnf ^+/−^* mice, in which the levels of BDNF in the brain are reduced by approximately half, and mice with an inducible deletion of *bdnf* in the forebrain exhibited blunted AD response in the forced swim test (Saarelainen et al., 2003; Monteggia et al., 2004). This result is related to the alteration of BDNF in the dentate gyrus of the hippocampus. Indeed, the selective deletion of BDNF in the dentate gyrus but not the CA1 region is sufficient to attenuate the effects of desipramine and citalopram in the forced swim test (Adachi et al., 2008). Further, in the BDNF^Met/Met^ mice, chronic fluoxetine is no more able to reverse stress-related behavior (Chen et al., 2006; Yu et al., 2012), to increase hippocampal BDNF levels and to stimulate dentate gyrus synaptic plasticity (Bath et al., 2012). It is, however, to note that the action of desipramine is still present (Yu et al., 2012), which suggests a specific contribution of the 5-HT system in these effects. These findings are coherent with clinical findings as it was observed that serum levels of BDNF were low in MDD patients and this normalizes after remission (Molendijk et al., 2011). The molecular target of BDNF is TrkB, and mice with a conditional deletion of the TrkB gene restricted to the forebrain do not respond to ADs (Saarelainen et al., 2003). Similar results were observed in Aquaporin-4 (AQP4) knockout animals. AQP4 is a key molecule for maintaining water homeostasis in the CNS that is expressed in adult neural stem cells and astrocytes. AQP4 invalidation disrupts the chronic fluoxetine-induced enhancement of adult mouse hippocampal neurogenesis (which is also a process crucially involved in the AD response; see next section) as well as AD-evoked behavioral improvement under both basal conditions and a chronic, mild stress-evoked depressive state (Kong et al., 2009).

Stress induces changes in the brain that can persist for the lifespan. Variations in genes implicated in 5-HT neurotransmission may interact with environmental factors to influence AD response (El-Hage et al., 2009). One group of signaling pathways involved in the cellular stress response includes the family of mitogen-activated protein kinases (MAPKs). Bruchas et al. (2011), focusing on the dorsal raphe nucleus, a brain region in which corticotropin-releasing factor, kappa-opioid receptors, and 5-HT systems converge, demonstrated that social defeat stress causes an increase in the activity of the intracellular signaling molecule p38α MAPK. They demonstrated that p38α MAPK activation within the dorsal raphe nucleus is responsible for the ability of stress to trigger depressive-like states. They demonstrated that in 5-HT neurons, p38α MAPK acts to directly influence SERT trafficking and ultimately increase the rate of 5-HT reuptake.

Another key molecular player in the response to monoaminergic ADs is p11. Indeed, p11 is downregulated in several brain regions of MDD patients or in animal models of MDD (Svenningsson et al., 2006; Alexander et al., 2010), whereas ADs and ECT increase p11 in the frontal cortex and hippocampus (Svenningsson et al., 2006; Warner-Schmidt et al., 2010; Oh et al., 2013). This response appears to be related to BDNF because p11 is reduced in mice in which BDNF is downregulated and increased in mice in which BDNF is overexpressed (Warner-Schmidt et al., 2010). p11 is also regulated by glucocorticoids (Zhang et al., 2008) and pro-inflammatory cytokines (Warner-Schmidt et al., 2011). Mice that lack p11 throughout their body display a depressive-like phenotype (Svenningsson et al., 2006; Warner-Schmidt et al., 2009, 2010), and more interestingly, the response to AD drugs is reduced in these mice (Svenningsson et al., 2006; Egeland et al., 2011; Eriksson et al., 2013). Interestingly, the accumbens-restricted overexpression of p11 is sufficient to induce the depressive-like phenotype in mice but does not modify the response to ADs (Alexander et al., 2010), whereas ablation of p11 from pyramidal projection neurons in the cortical layer 5A does not alter depressive-like behavior but results in a diminished response to ADs (Schmidt et al., 2012a), indicating that the mechanisms underlying the pathophysiology of MDD might differ from the mechanisms underlying the response to ADs.

Recent studies have provided evidence for a *BDNF* gene × environment interaction. Stress and ADs have opposing actions on BDNF and neurogenesis. Stress decreases and ADs increase the expression of BDNF in the dentate gyrus granule cell layer. These changes contribute to the regulation of neurogenesis by stress and ADs (Duman and Li, 2012). Mutant mice with a heterozygous deletion of BDNF, which results in the expression of approximately half the normal levels of BDNF, display normal behavior under baseline conditions but exhibit a depressive phenotype upon exposure to stress and an altered response to ADs (Duman et al., 2007; Ibarguen-Vargas et al., 2009).

Other factors have been implicated in the response to AD (e.g., zinc and cytokines). Clinical evidence suggests that zinc deficiency induces depression- and anxiety-like behaviors. Zinc administration improves the efficacy of ADs in MDD patients and may be particularly relevant for TRD patients. Recent investigations on the molecular mechanisms responsible for these observations suggest a role for zinc in the regulation of neurotransmitters, antioxidant mechanisms, neurotrophic factors and neuronal precursor cells. The presynaptic release of zinc from axon terminals of glutamatergic neurons is prominent in the hippocampus, where zinc exerts complex pleiotropic effects on neuronal plasticity, neurogenesis, and neuronal viability, affecting learning, memory, and emotional regulation (Swardfager et al., 2013). The neuroprotective properties of zinc at physiological concentrations may be attributable to the blockade of excitotoxic Ca^2+^ in?ux and upregulation of cellular antioxidant systems. Chronic zinc administration can increase BDNF expression (Swardfager et al., 2013).

Conboy et al. (2011) explored the expression of macrophage migration inhibitory factor (MIF) in astrocytes and neurogenic cells in the subgranular zone of the rodent dentate gyrus and characterized its presence in stem cells, cells undergoing proliferation, and recently proliferated cells undergoing maturation. They found that MIF deficiency is associated with a phenotype characterized by increased anxiety- and depression-like behaviors. Furthermore, they determined that in the subgranular zone of the hippocampus, macrophage MIF expression is modulated in parallel with cell proliferation by stress, glucocorticoid levels, and fluoxetine. Both the genetic deletion of MIF and chronic treatment with the MIF antagonist Iso-1 resulted in reduced cell proliferation, and MIF deletion also abolished the enhanced proliferation induced by chronic fluoxetine.

Moon et al. (2012) provided evidence that macrophage MIF is regulated by long-term exercise and that it mediates induction of serotonin and neurotrophic factors, resulting in the amelioration of depressive behaviors in a rodent model. MIF was upregulated during both long-term voluntary exercise and repeated electroconvulsive seizure treatments. The authors demonstrated that MIF induced *TPH2* and *Bdnf* expression in the rat brain along with ERK1/2 activation, resulting in an increase in 5-HT levels by MIF. In addition, direct intra-brain administration of MIF induced an AD-like response in the forced swim test. These results demonstrate that MIF induces AD-like behavior and mediates the effect of exercise on mood improvement (Moon et al., 2012). Physical activity has neuroimmune effects that are likely involved in enhanced neuroplasticity, reduced oxidative stress, increases in 5-HT, dopamine, and noradrenaline, and enhanced glucocorticoid sensitivity (Eyre et al., 2013). Thus, physical activity should be recommended in addition to AD therapy to improve drug response.

#### Cellular targets

In the past 15 years, the process leading to the generation of new neurons in the adult hippocampus (adult neurogenesis) has been shown to be compromised in rodent models of MDD; this effect is prevented by administration of monoaminergic AD drugs (see Hanson et al., 2011; Eisch and Petrik, 2012; Petrik et al., 2012; Bambico and Belzung, 2013; Tanti and Belzung, 2013a,b for recent reviews). These data have been corroborated by clinical studies (Boldrini et al., 2009, 2012, 2013), although some contradictory findings have been initially reported (Reif et al., 2006). Furthermore, these data extend beyond pharmacotherapy, as increase in hippocampal neurogenesis has also been observed after ECT (Malberg et al., 2000) or VNS (Revesz et al., 2008). However, increase in adult hippocampal neurogenesis alone is insufficient to induce AD-like effects (Sahay et al., 2011). Interestingly, studies have established that the ability of AD treatments to stimulate neurogenesis applies to all phases of the generation of new cells (proliferation, maturation) and is observed in the ventral and dorsal hippocampus (Tanti and Belzung, 2013b). Furthermore, adult hippocampal neurogenesis is not only a correlate of the therapeutic action of monoaminergic drugs but appears to be essential to achieve recovery. Indeed, abolition of hippocampal neurogenesis by focal X-ray hippocampal irradiation suppresses some and/or all behavioral effects of fluoxetine and/or imipramine in animals (Santarelli et al., 2003; Airan et al., 2007; Surget et al., 2008, 2011; Wang et al., 2008b; David et al., 2009; Perera et al., 2011). Similar results are observed after genetic ablation of adult neurogenesis (Lehmann et al., 2013). However, the situation is more complicated if we consider the effects of newly developed putative ADs: indeed, whereas the effects of a CNR1 ligand are abolished by suppression of neurogenesis (Jiang et al., 2005) as observed with monoaminergic-acting compounds, most of the AD-like effects of MCHR1 (Melanin-concentrating hormone receptor 1), CRH1 (corticotrophin-releasing hormone), or V1b (Vasopressin V1b) antagonists are not prevented by ablation of neurogenesis (David et al., 2007; Surget et al., 2008). This result indicates that although the ability of the treatments used in the clinic (which all target monoaminergic systems) to elicit remission relies on neurogenesis, the therapeutic effects of a drug can also be achieved via neurogenesis-independent mechanisms. For example, the dual orexine receptor almorexant induces AD-like effects but can decrease neurogenesis in some cases (Nollet et al., 2012), and rTMS suppresses the survival rate of proliferating cells (Czéh et al., 2002). The ability of new cells to contribute to recovery requires the incorporation of the new neurons into a functional network, which occurs when these cells are 4–8 weeks old. Thus, the use of an anti-mitotic agent to suppress neurogenesis revealed that the ablation of 2-week-old cells did not modify the effects of ADs (Bessa et al., 2009), whereas the 4-week-old cells are recruited after successful AD therapy to facilitate the regulation of the HPA axis after disruption by chronic stress (Surget et al., 2011). Therefore, it appears that hippocampal neurogenesis can be considered a process underlying AD non-response, related to the generation of new functional neurons.

The essential role of adult hippocampal neurogenesis in achieving remission is further supported by the observation that environmental factors that contribute to remission also act on adult hippocampal neurogenesis. Indeed, environmental enrichment and running both enable recovery and stimulate neurogenesis. Furthermore, the AD-like effects of environmental enrichment are suppressed by genetic abolition of neurogenesis (Schloesser et al., 2010). Factors associated with higher AD non-response, such as aging, are also related to lower hippocampal neurogenesis (Couillard-Despres et al., 2009).

Several hypotheses have been formulated regarding the function of adult hippocampal neurogenesis. According to some authors, this process might be crucial for pattern separation (Clelland er al., 2009; Sahay et al., 2011; Nakashiba et al., 2012; Tronel et al., 2012), a process that enables the differentiation of two closely overlapping stimuli. Thus, a deficit in pattern separation might lead to overgeneralization and cognitive bias. A restoration of such a deficit could contribute to remission. Others claim that hippocampal neurogenesis is required for contextual memory (Shors et al., 2001; Dupret et al., 2008; Deng et al., 2009; Trouche et al., 2009). Hippocampal neurogenesis is essential for regulation of the HPA axis (Schloesser et al., 2009; Snyder et al., 2011; Surget et al., 2011), and an increase in neurogenesis might facilitate recovery via normalization of HPA axis function. Finally, hippocampal neurogenesis have also been proposed to be crucial for anxiety behavior (Revest et al., 2009; Fuss et al., 2010; Mateus-Pinheiro et al., 2013) or executive functions (Burghardt et al., 2012); if restored, these functions would enable the subject to react more accurately to the environment. Interestingly, all of the above-mentioned processes are disturbed in MDD and could be essential to achieving remission (see Tanti and Belzung, 2013a,b, for reviews).

If hippocampal neurogenesis is crucial in achieving remission, any strategy that leads to an increase in the number of new neurons in this structure could facilitate recovery or accelerate the onset of therapeutic outcome when combined with ADs. Several processes have been shown to increase neurogenesis, including environment enrichment, physical exercise, and learning. Thus, one can speculate that combining any of these activities with AD treatment could increase the therapeutic outcome of pharmacotherapy.

### Hormonal targets and response to antidepressants

#### HPA axis regulation

It has been repeatedly demonstrated that ~50% of patients with MDD exhibit a dysfunction of the tuning of the HPA axis (Young et al., 1991; Holsboer, 2000; Pariante and Miller, 2001; Pariante, 2003), which can be measured using the dexamethasone-suppression test or combined dexamethasone/CRH test. Indeed, these patients exhibit dexamethasone non-suppression, indicating a defect in the negative feedback that enables the suppression of the secretion of glucocorticoids in healthy subjects. This dysregulation is restored after effective AD therapy, and interestingly, the therapeutic action of the treatment only occurs once normal feedback of the HPA axis has been restored (see Belzung and Billette De Villemeur, 2010, for a review), indicating that restoration of the HPA axis regulation is mandatory to enable recovery. Interestingly, normalization of HPA axis overactivity also occurs after successful ECT (Kling et al., 1994), indicating that this property goes beyond classical pharmacotherapy. Thus, the tuning of the HPA axis may be related to the ability to achieve a therapeutic outcome after treatment. If this outcome cannot be achieved, a resistance and/or non-response to treatment should be observed (Ising et al., 2007; Binder et al., 2009; Hennings et al., 2009; Schüle et al., 2009; Horstmann et al., 2010). This relationship has been demonstrated: a defect in HPA axis regulation is not only a signature of MDD or of remission but also a predictor of treatment non-response. Indeed, using the combined dexamethasone/CRH test, Brouwer et al. (2006) demonstrated that the AD response rate in high-ACTH patients was significantly lower than that in intermediate-ACTH patients. Similar results have been reported by Ising et al. (2007), who demonstrated that patients who do not exhibit a reduction of the cortisol response to a dexamethasone/CRH test after 2–3 weeks of treatment are not likely to respond to the current treatment. However, this has not been confirmed in two other studies (Paslakis et al., 2010; Carpenter et al., 2011). For example, Carpenter et al. (2011) rather found an increased pre-treatment cortisol after DEX/CRH to be associated with sertraline response. Possibly, rapid initial improvement of the cortisol response following DEX/CRH would be a more effective predictor of response outcome.

At the molecular level, the negative feedback on the HPA axis mainly occurs via the binding of glucocorticoids, such as cortisol or corticosterone, on specific receptors, such as the glucocorticoid receptors (GRs), which have low affinity for glucocorticoids and are thus only activated when a high level of stress hormones is present. The mode of action of GRs is very complex: in the absence of glucocorticoids, GRs are packaged into a large molecular complex consisting of chaperone heat-shock proteins, such as *hsp90* and *hsp70*, and co-chaperones, such as *FKBP51*, which bind to the receptor and keep it inactive, thus decreasing the affinity of GRs for glucocorticoids. High *FKBP51* induces inhibition of glucocorticoid-related GR activation, and thus, *FKBP51* can be considered a negative feedback loop regulating GRs (Schmidt et al., 2012b). Interestingly, in MDD subjects, peripheral *FKBP51* reduction is a marker for successful AD therapeutic outcome because FKBP51 levels are diminished in AD responders but not non-responders(Cattaneo et al., 2013).

The contribution of HPA regulation to treatment outcome after AD has been confirmed by studies investigating the contribution of genes involved in the tuning of the stress axis after treatment. Most findings concern the *FKBP5* gene. Several reports have investigated the association of *FKBP5* polymorphisms with the response to AD drugs. In 2004, a strong association between polymorphisms of the *FKBP5* gene and the response to AD was observed in 280 depressed patients of the MARS sample (Binder et al., 2004). These results were subsequently replicated in the STAR^*^D sample (Lekman et al., 2008) as well as in another German sample (Kirchheiner et al., 2008). Interestingly, this relationship appears to be independent of the class of AD drug because it was observed in patients treated with TCAs, SSRIs, or mirtazapine. However, two smaller studies of Spanish and Korean subjects reported negative associations in small samples (Papiol et al., 2007; Tsai et al., 2007). In fact, in the patients carrying the genotypes associated with faster response to AD, pre-treatment HPA-axis dysregulation was low compared to other patients (Binder et al., 2004), which might have facilitated the normalization of the HPA-axis and thus recovery.

Polymorphisms of the *GR* gene have also been studied in association with treatment outcome. Studies have demonstrated that the BclI and ER22/23EK polymorphisms are associated with the response to ADs (Brouwer et al., 2006; van Rossum et al., 2006). Other association studies have focused on the influence of polymorphisms in the *CRHR1*, *CRHR2*, and *CRH-BP* genes. Allele G carriers at the rs2270007 site of the *CRHR2* gene exhibited a worse response to the SSRI citalopram (Papiol et al., 2007) when compared to other alleles. Similarly, in a Chinese population, polymorphism at the rs242941 site of the *CRH1* gene has been associated with fluoxetine response (Liu et al., 2007b). Finally, polymorphisms of genes related to hsp70 protein have also been associated with poor response to AD (Pae et al., 2007a). Taken together, these genetic studies strengthen the view that a pre-treatment defect in HPA axis regulation may predict poor treatment outcome.

Environmental studies further corroborate this assertion. Hyperactivity of the HPA axis is related to chronic stress. Interestingly, two recent publications reported that elevated stress prior to treatment was associated with a good response to SSRIs (Keers et al., 2010; Horacek et al., 2011). However, other studies have shown the opposite (Monroe et al., 1992) or no association (Bock et al., 2009). These discrepancies could be related to gene × environment interactions because the effects of stress on treatment outcome may vary according to genotype. For example, in patients carrying more than one *s* allele of the *SERT* gene (which is associated with increased vulnerability to stress), the occurrence of a stressor predicted a poor response to ADs (Mandelli et al., 2009; Keers et al., 2011). Similar findings were obtained using a large sample of patients treated with escitalopram; in patients carrying polymorphisms inducing vulnerability to stress, such as polymorphism at the rs1360780 site of the *FKBP5* gene and at the rs110402 site of the *CRHR1* gene, stressors were predictive of response to treatment (Keers and Uher, 2012).

Finally, evidence for the involvement of HPA axis feedback dysregulation in AD non-response is also provided by data from patients with diseases that are highly comorbid with MDD, such as Cushing's disease. Cushing's disease is a syndrome related to chronic glucocorticoid excess, due either to an overproduction of ACTH caused by a pituitary adenoma (66% of patients), to an adrenal adenoma or carcinoma (24% of patients), or to ectopic causes (10% of patients). Approximately half of the patients display MDD (Sonino and Fava, 2002; Pereira et al., 2010). Interestingly, MDD patients with Cushing's disease are poor responder to treatment with classical AD therapy (Sonino et al., 1986, 1993), whereas a good therapeutic outcome can be achieved by treatments that normalize the HPA overactivity (Sonino and Fava, 2002; Pereira et al., 2010). These results confirm that excessive function of the HPA axis is associated with AD non-response.

To improve drug response, pre-treatment HPA dysfunction should be assessed with the dexamethasone suppression test or, preferably, the combined dexamethasone/CRH, as this latter test appears to be more specific (Ising et al., 2007). Polymorphisms of HPA-related genes, such as the *FKBP5*, *CRHR1*, or GR genes, would also be highly relevant, even if they are not yet currently used in clinical practice. If these markers predict poor HPA function, one could predict that treatments targeting the HPA axis (CRF1 antagonists, V1b receptor antagonists, GR antagonists, FRBP51 inhibitors) could be useful therapeutic strategies to improve drug response as well as the onset of response when combined with more conventional ADs.

#### Thyrotropin releasing hormone (TRH)

The relationship between thyroid function and MDD is well-known. Indeed, hypothyroidism is associated with MDD, and TRH levels correlate with symptom severity (Bauer et al., 2009). Furthermore, MDD patients exhibit alterations in several markers of thyroid function, particularly of the thyroid hormone triiodothyronine (T3) (see Hage and Azar, 2012 for a review). Interestingly, T3 is widely used to improve the therapeutic outcome of AD drugs in patients who exhibit poor response to treatment (Aronson et al., 1996; Nierenberg et al., 2006), particularly in those treated with TCAs. Successful treatment with T3 alone has also been reported in some older studies (Feldmesser-Reiss, 1958; Flach et al., 1958; Wilson et al., 1974). These findings are further supported by preclinical evidence. Indeed, T3 alone elicited an AD-like effect in a bio-assay for AD response (Lifschytz et al., 2006, 2011), whereas the combination of T3 with chronic fluoxetine increased the effects of the SSRI (Brochet et al., 1987; Eitan et al., 2010). This effect appears to be related to thyroid function because it has also been shown that the effects of TCAs are reduced in rats in which hypothyroidism has been provoked by the addition of propylthiouracil to their drinking water (Martin et al., 1987). The effects of T3 occur via an interaction with nuclear thyroid hormone receptors (TRs). Four such receptors have been described: α-1, α-2, β-1, and β-2. It would be of interest to determine which of these receptors might mediate the effects of T3. A recent study demonstrated that administration of dronedarone, a specific TRα antagonist, prevented the AD-like effects of T3, suggesting that the TRα receptor is responsible for the effects of T3 (Lifschytz et al., 2011). This observation is consistent with the finding that TRα receptors are the most highly expressed TRs in the brain (Williams, 2008) as well as with the observation that mice in which this receptor is mutated display depressive-like behavior (Pilhatsch et al., 2010).

Few studies have investigated the association of polymorphisms of thyroid function-related genes with MDD and AD response. However, the relationship of a polymorphism of the deiodinase type I (*D1*) gene (D1 converts inactive T4 to active T3) with the ability of T3 to augment the effects of AD has been investigated (Papakostas et al., 2009).

Interestingly, the effects of T3 in patients undergoing AD therapy are particularly remarkable in some subcategories of patients, particularly in women (Altshuler et al., 2001; Agid and Lerer, 2003) and in patients with atypical MDD.

Further progress could certainly be achieved in improving AD response by (a) dosing pre-treatment T3 hormones in patients and (b) studying polymorphisms of thyroid function related genes. Such studies would enable the segregation of patients according to their thyroid hormone status and the initiation of co-therapy with T3 at the beginning of AD therapy in those patients with the highest thyroid dysfunction.

## Discussion

To summarize, we know from various evidence from the litterature that response to ADs can be driven but also altered at various levels (Table 1). Derived from these data, several peripheral or central biomarkers can now enable predicting an increased risk of non-response to AD treatment. They include markers genetic testing (polymorphisms of *P450, ABCB1, SERT, NERT, COMT, MAOA, leptin, FKBP5, hsp70, GR, BclI, CRHR1, CRHR2, BDBF, D1, TR*α genes, etc), plasmatic dosage (BDNF, cortisol, FKBP51, etc.) or brain imaging. Predictors of treatment response can easily be uncovered by deduction from predictors of treatment non-response: for example, if a poor response to ADs is met in Val allele carriers of the BDNF gene, this also indicates that good response might be achieved in the Met carriers. If poor response is shown in patients having low rostral cingulate activity, high response is observed in patients exhibiting elevated pretreatment rostral cingulate activity.

**Figure 1:**
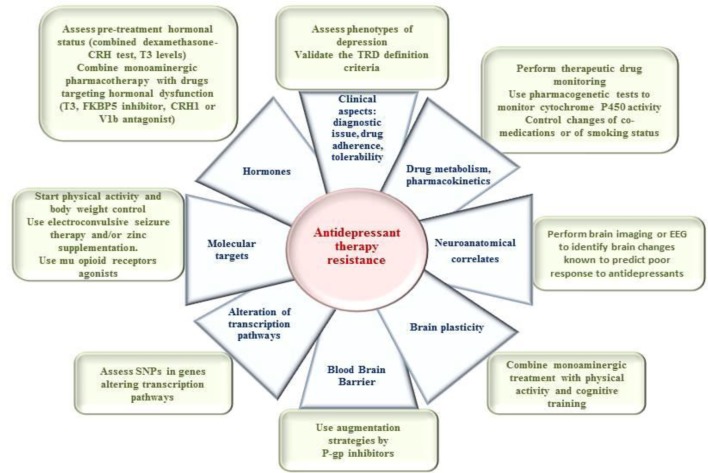
**Mechanisms (in blue) associated with antidepressant therapy resistance and recommendations for clinical practice (in green)**.

Based on the potential mechanisms of response to AD, several practical implications may be deducted (see Figure 1). Some of these conclusions have entered clinical routine and are now part of practical recommendations, such as augmentation therapy by thyroid hormones, and are sustained by strong clinical evidence (Shelton et al., 2010). However, other innovative strategies may enter further clinical investigation through randomized controlled studies. Among these new strategies, controlling co-medications, smoking status, or weight, assessing pre-treatment hormonal status combined with dexamethasone/CRH tests and T3 levels, investigating SNPs of specific genes known to be implicated in AD non-response, and the use of brain imaging or EEG to identify brain changes known to predict poor response to ADs are among the most promising. A more systematic referral to therapeutic drug monitoring (plasma concentration quantification) would also be useful as it appears to be largely insufficiently used given the considerable inter-individual variability in the pharmacokinetic characteristics of drugs. It enables the adaptation of the dosage of ADs to achieve the plasma drug concentration that ensures the highest probability of response (Hiemke et al., 2011). New augmentation strategies could also be developed based on the evidence reported in the present review, such as combining monoaminergic treatment with physical activity, cognitive training, stress reduction, P-gp inhibitors, mu opioid receptors agonists, zinc, and drugs targeting hormonal dysfunction (T3, FKBP5 inhibitor, or CRH1 or V1b antagonists). Switching ADs from one ineffective AD to a similar or different class of ADs and from SSRI/SNRIs to TCAs, MAOIs, and non-conventional antidepressant drugs, such as NMDA antagonists, may be other valuable strategies, as well as switching to somatic therapies, such as ECT, rTMS, VNS, or DBS.

One of the main obstacles to improving care strategies is the wide heterogeneity of patients labeled as suffering from TRD and/or showing insufficient response to conventional AD. This heterogeneity is the consequence of the relatively poor specificity of the criteria for diagnosis, which are still based on clinical evaluation; even the rating scales typically used to assess clinical response exhibit relatively good inter-rater and test-retest validity. Moving toward a more systematic use of biomarkers may improve the characterization of clinical phenotypes of MDD and their biological, imaging or genetic, proteomic and metabolomic correlates (Leuchter et al., 2010). As we have extensively reviewed, although TRD and/or treatment non-response is a considerable challenge to improving patient outcome and preventing severe complications of prolonged depressive states, it represents a unique opportunity to better understand mechanism of action of ADs and thus to better understand the pathophysiology of MDD and improve its clinical characterization. This potential makes research on TRD and/or treatment non-response a high priority for new research developments at both the preclinical and clinical levels.

### Conflict of interest statement

The authors declare that the research was conducted in the absence of any commercial or financial relationships that could be construed as a potential conflict of interest.
